# Knowledge Retention From Emergency Medicine Simulation-Based Learning Curriculum for Pre-clinical Medical Students

**DOI:** 10.7759/cureus.41216

**Published:** 2023-06-30

**Authors:** Jennifer C McMains, Michael C Larkins, Alexandra M Doherty, Julia Horiates, Kamel Alachraf, Julian A Gordon, James Fletcher, Kori L Brewer

**Affiliations:** 1 Emergency Medicine, East Carolina University Brody School of Medicine, Greenville, USA

**Keywords:** interprofessional education and collaboration, interdisciplinary simulation, simulation in medical education, teaching in emergency medicine, simulation based learning

## Abstract

Introduction

Traditional medical school curricula rely on textbook-based learning during the first two years, often limiting students' clinical exposure. Simulation-based learning (SBL) provides an opportunity for students to gain clinical exposure and competency with common procedures as well as to gain knowledge related to common clinical topics. Retention of factual knowledge is a current topic of discussion as medical learners often have difficulty with long-term retention. The aim of this study was to assess if students would learn, retain, and enjoy emergency medicine (EM)-focused SBL.

Materials and methods

We developed an EM-focused SBL curriculum consisting of four main educational events: suturing, medical stabilization, mass casualty triage, and point-of-care ultrasound (POCUS). Participants were first- and second-year healthcare students enrolled in a traditional, preclinical curriculum, who completed pre- and post-event quizzes consisting of multiple-choice questions on topics covered during the SBL scenario. We compared pre- and post-event quiz scores using a one-way paired t-test. Quizzes were readministered up to 100 days after each SBL event to test knowledge retention, and scores were compared across time by repeated-measures analysis of variance (RMANOVA).

Results

For suture (n=22), mass casualty (n=20), and ultrasound simulations (n=17), post-event mean quiz scores increased significantly in comparison to mean quiz scores from before the event (p≤0.05). Medical stabilization simulation post-event scores were increased but did not reach statistical significance. Data collected at 45, 74, and 94 days following the suture lab as well as 29 and 49 days after the medical evacuation event, and 20 days after the mass casualty event showed no statistical decrease in quiz means suggesting retention of knowledge among learners. Subjective assessments of participant satisfaction demonstrated an enjoyment of the events.

Discussion

EM-focused SBL events offered enjoyable learning opportunities for students to effectively obtain and possibly retain clinical knowledge.

Conclusion

SBL has the potential to improve student retention of clinical knowledge during the preclinical years and, therefore, should be further explored and implemented as a core pillar of medical education as opposed to its current state as a learning adjunct.

## Introduction

Simulation-based learning (SBL) continues to increase in popularity in medical education, ranging from simulations of resuscitation scenarios for medical students on their first day, to resident physicians practicing central venous catheters, to multi-disciplinary teams practicing communication during primary and secondary assessments of trauma patients [1-3]. Prior studies have shown that exposure to SBL, even in undergraduate education, can increase clinical success specifically regarding technical skills such as CPR and airway management [4]. These benefits have been enhanced with advances in simulation technology [5-8]. Meta-analyses of SBL across more than 100 different sources indicate that SBL is viewed favorably among participants and greatly enhances the acquisition of technical and manual skills [5,6].

While the acquisition of skills is important, the acquisition of knowledge is a key focus during medical school. Beyond just knowledge acquisition, knowledge retention is a concept gaining increased focus in graduate medical education as students enrolled in medical school are required to learn a large amount of information quickly; this is colloquially known as “drinking water from a fire hydrant.” The reality that medical students struggle to retain this large amount of information is an area of concern, as licensing exams and, more importantly, patient care rely on a physician’s quick recall of a wide range of topics [9]. In order to address these concerns, retention strategies such as spaced repetition and problem-based learning have been implemented [10]. SBL provides an opportunity for continued and improved knowledge retention in clinical areas. This study seeks to look beyond technical skill acquisition from SBL into the use of SBL as a method to increase the retention of factual knowledge regarding clinical topics.

We focused our SBL-based curriculum on the specialty of emergency medicine (EM). At many medical schools, students do not gain significant exposure to EM until their final year of study, a point at which students have often already chosen their desired specialty for residency training [11]. SBL if placed earlier in the medical curriculum can provide students with exposure to EM cases and procedures that students wouldn’t otherwise experience. Early exposure to SBL has been shown to increase students’ confidence and satisfaction with their medical education [12]. The objective of our study was to create an EM-focused SBL curriculum where students in the foundational years of medical school would have an opportunity to learn clinical information relating to EM far earlier than would usual and to assess if participants learned from, retained knowledge from, and enjoyed this curriculum.

## Materials and methods

Creation of curriculum

We created a short curriculum consisting of four SBL events: a suture lab, a medical evacuation (MedEvac) simulation, an Inter-professional Triage and Emergency, Assessment, and Management (ITEAM) day, and a point-of-care ultrasound (POCUS) lab. This curriculum was implemented in the fall of 2021. Each event had a similar schedule: a pre-event test followed by a brief presentation introducing the topic, then the simulation, followed by a post-event test and enjoyment survey. At the consecutive events, students completed retention assessments. We grouped students into “attendee” vs “non-attendee” in order to assess retention data. Students were not permitted to utilize any external resources during assessments. All data were collected anonymously and securely stored.

The pre- and post-test consisted of the same five to 10 objective multiple-choice questions regarding the chosen clinical topics. These questions were to assess factual knowledge as opposed to technical skills. A group of EM-attending physicians created these multiple-choice questions, while other senior-level physicians reviewed these assessments. The same questions were utilized in both pre- and post-event assessments, as well as at subsequent events to assess the retention of acquired knowledge. Physician facilitators were given creative licenses for what concepts they presented and the methodology of presentation prior to the SBL event. We provided a copy of the knowledge assessment to facilitators to ensure they discussed these key concepts. Additionally, facilitators worked with authors and staff at the Office of Clinical Simulation to create individual simulation events. Details of the simulation setup are provided in the Appendix. We also created a survey to assess students’ perceptions of enjoyment and efficacy of these events. Students were able to rate their enjoyment of the SBL event and interest in future SBL events by responding to questions with a 5-point Likert scale, wherein a one indicated “strongly disagree” and a five indicated “strongly agree.”

An EM attending and five resident physicians from various specialties facilitated the suture lab. Students completed the pre-event test immediately prior to the event. Following this, facilitators taught students simple interrupted and continuous suture techniques and the indications for each through verbal explanation followed by demonstrating the techniques to small groups of students. Students were then given the opportunity to practice these techniques with simulated suture materials. At the end of this event, students completed the post-event test and enjoyment survey.

At the MedEvac event, students first completed the MedEvac pre-event test and the suture retention test. Then, three EM attending physicians discussed medical stabilization skills before splitting into small groups where students practiced performing primary surveys, utilizing airway adjuncts, using bag valve masks, and executing circulatory compromise management with tourniquets. At the end of this event, students completed the MedEvac post-event test and enjoyment survey.

Over the course of a half-day, eight EM attending and resident physicians, one physician assistant (PA), and one nurse practitioner facilitated the ITEAM day event where students learned from simulation scenarios including primary survey, intraosseous and intravenous line placement, hemorrhage management, organophosphate decontamination, airway placement, and the initiation of care during the preliminary phase of cardiopulmonary arrest. Students also engaged in a simulated mass casualty event involving the triage of over 20 standardized patients. Standardized patients were trained to emulate survivors of a mass casualty, with various scripted behaviors and wounds of varying severities. At the beginning of this event, students completed not only the ITEAM pre-event test but also the retention post-event test regarding the suture and MedEvac events. At the end of this event, students completed ITEAM post-event test and enjoyment survey.

Lastly, an EM attending and four resident physicians taught students basic ultrasound physics and knobology as well as POCUS indications and techniques including assessing function and deformity of the heart, lungs, liver, spleen, and bladder which was then simulated via standardized patients. Immediately prior to the simulation, students completed not only the ultrasound pre-event test but also the retention post-event test for the suture, MedEvac, and ITEAM events. Immediately following the event, students completed the ultrasound post-event test and enjoyment survey.

We recruited first- and second-year medical students at a rural public medical school to engage in these educational activities. At the ITEAM day event, PA students were also included. Participants less than 18 years old or those not enrolled in the medical or PA school were excluded from this study. We publicized these events to students via email listservs. Students voluntarily signed up to participate in each event; therefore, not every student participated in all events. This study was certified exempt by East Carolina University and Medical Center Institutional Review Board, study number 21-001721, entitled "Efficacy of Extracurricular Simulation-Based Learning for Medical Students in Didactic Years."

Data analysis

We exported de-identified survey responses from a browser-based electronic survey into Microsoft Excel (Microsoft, Washington, USA), and subsequent statistical analysis was performed. For all simulation events, pre- and post-scores on individual knowledge assessments were compared using a one-way paired t-test with p<0.05 indicating significance (JMP v.15 pro; SAS, Inc.).

We assessed retention of knowledge over time using repeated-measures analysis of variance (RMANOVA) with p<0.05 indicating significance (JMP v.15 pro; SAS, Inc.). Knowledge scores obtained immediately after each simulation event (post-event) were compared to those obtained at the following intervals: 20 to 45 days, 45 to 70 days, and greater than 90 days after the corresponding initial event. Knowledge scores obtained during the suture simulation were measured at all four time points, MedEvac at three time points, and ITEAM at two time points. Ultrasound knowledge scores were only measured pre- and post-simulation, as follow-up events were canceled due to changing COVID regulations.

All statistics are reported as mean ± standard error of mean (SEM) with 95% confidence intervals (CI). The difference between the means and the 95% CI of the difference is also reported where appropriate. Data from enjoyment surveys are reported as descriptive data including mean, median, and mode.

## Results

Knowledge acquisition from simulation events

Twenty-two healthcare students completed both pre- and post-event quizzes for the suture simulation, 15 for the MedEvac simulation, 20 for the ITEAM simulation, and 17 for the ultrasound simulations. For suture, ITEAM, and ultrasound simulations, knowledge scores showed significant improvement after the event in comparison with scores obtained before the event (p≤0.05; Table 1). Scores for the MedEvac simulation approached significance (p=0.06), but this comparison was underpowered (56%) due to the small sample size.

**Table 1:** Pre- and post-SBL event knowledge assessment scores MedEvac: medical evacuation simulation event, ITEAM: inter-professional triage and emergency, assessment, and management event, SEM^a^: standard error of the mean, CI: confidence interval

Event	Pre-event Score Mean ± SEM^a^ [95% CI]	Post-event Score Mean ± SEM^a^ [95% CI]	p-value
Suture (N = 22)	34.5 ± 5.5% [23.2, 45.9]	73.6 ± 5.2% [62.9, 84.4]	<0.001
MedEvac (N = 15)	54.4 ± 6.6% [40.2, 68.6]	70.8 ± 5.4% [59.4, 82.3]	0.06
ITEAM (N = 20)	72.5 ± 5.7% [60.6, 84.4]	91.7 ± 2.4% [86.5, 96.8]	0.005
Ultrasound (N = 17)	31.3 ± 3.5% [23.6, 38.5]	56.1± 6.7% [41.5, 70.8]	0.004

Knowledge retention from simulation events

Regarding retention, the responses directly after the event and then at 20-45 days after the event, 45-70 days after the event, and last more than 90 days after the initial event showed no statistical decline in scores over time (Table 2). For the suture lab, assessment scores were similar when taken immediately following (n=22), at 45 days (n=11), at 74 days (n=7), and at 94 days (n=11) after the initial event. For the medical evacuation SBL event, there was also no significant decreased in scores from the post-event survey (n=15) to the 29 day time point (n=6), to 49 days after the original event (n=8). Lastly for ITEAM day, there was no significant decrease in scores from the event (n=20) to 20 day after the event (n=6). Due to COVID regulations, events planned to assess retention of the ultrasound event were canceled, and, therefore, retention from the ultrasound SBL event was not assessed.

**Table 2:** SBL event knowledge retention scores MedEvac: medical evacuation simulation event, ITEAM: inter-professional triage and emergency, assessment, and management event, SEM^a^: standard error of the mean, CI: confidence interval N values for retention of suture knowledge were 22, 11, 7, and 11 at post-event, 45 days after, 74 days after, and 94 days after, respectively. N values for retention of medical evacuation knowledge were 15, 6, and 8 at post-event, 29 days after, and 49 days after, respectively. N values for retention of mass casualty knowledge were 20 and 6 at post-event and 20 days after, respectively.

Event	Post-event Score: Mean ± SEM^a^ [95% CI]	20-45-Day Retention: Mean ± SEM^a^ [95% CI]	45-75-Day Retention: Mean ± SEM^a^ [95% CI]	>90-Day Retention: Mean ± SEM^a^ [95% CI]	p-value
Suture	73.6 ± 5.2% [62.9, 84.4]	60.0± 9.7% [38.3, 81.7]	57.5 ± 11% [30.1, 84.2]	60 ± 7.1% [44.1, 75.9]	0.31
MedEvac	70.8 ± 5.8 [60.0, 82.7]	80.6 ± 9.4 [61.2, 99.9]	58.3 ± 8.2 [41.6, 75.1]	-	0.26
ITEAM	91.7± 5.2% [81.1, 100]	75.0 ± 9.1 [56.7, 93.3]	-	-	0.21

Enjoyment of simulation events

Student enjoyment and self-perceived learning from all simulation events were assessed using a 5-point Likert scale, with a five indicating “strongly agree” and a one indicating “strongly disagree.” These results can be seen in Table 3, with the survey question in the leftmost column and the responses in each corresponding column to the right. In summary, students enjoyed the events and strongly agreed that they enjoyed the event more than a lecture on similar topics. Furthermore, students subjectively felt like they learned from these events as well.

**Table 3:** Student enjoyment of SBL ^a^ Rated on a 5-point Likert scale (1 = strongly disagree, 5 = strongly agree) MedEvac: medical evacuation simulation event, ITEAM: inter-professional triage and emergency, assessment, and management event

Enjoyment Metric	Suture (N = 22)	MedEvac (N = 17)	ITEAM (N = 19)	Ultrasound (N = 15)
“I enjoyed this event.” Mean (Median)^a^	5.00 (5)	5.00 (5)	4.89 (5)	5.00 (5)
“I learned from this event.” Mean (Median)^a^	5.00 (5)	4.94 (5)	5.00 (5)	4.93 (5)
“I enjoyed learning from this event more than I would have enjoyed learning the same content from a lecture.” Mean (Median)^a^	5.00 (5)	5.00 (5)	4.89 (5)	4.93 (5)
“I learned more effectively from this event than I would have learned the same content from a lecture.” Mean (Median)^a^	4.90 (5)	5.00 (5)	4.83 (5)	4.86 (5)
“I would attend a similar event like this again.” Mean (Median)^a^	4.95 (5)	5.00 (5)	4.94 (5)	5.00 (5)

## Discussion

This four-part EM-focused SBL curriculum is an effective instruction methodology for healthcare students during their didactic years, as supported by this study. Medical students who had not yet begun their clinical education were able to quickly learn both clinical skills and related knowledge. More importantly, the data shows no significant decline in scores over time which can be interpreted as meaningful knowledge retention in these students following the SBL events. This continued retention is likely due to two main reasons: the clinical knowledge was presented in a way to build on previous knowledge and the SBL event optimized the use of active and kinesthetic learning.

These SBL events provided scenarios in which students could apply and build upon basic scientific knowledge obtained from lectures. It has been shown that students retain new knowledge more effectively when they are able to attach a clinical scenario to factual knowledge to create layers of knowledge creating a deeper and more permanent state of understanding [13]. Through these SBL events, students were able to participate in clinical scenarios in which didactic learning became clinically relevant.

The ability of SBL to provide exposure to EM has become even more important following the state of emergency declared in March 2020. As a response to the emerging COVID-19 crisis, many teaching hospitals canceled clinical clerkships while also moving the majority of education online [14]. Over time, third- and fourth-year students were slowly reintroduced into the clinical space when it was deemed safe enough to do so. However, for many first- and second-year students, clinical experiences were still limited or prohibited. There has been much discussion regarding healthcare programs utilizing SBL as a clinical alternative during the pandemic [15,16]. SBL events provide a method through which students in their didactic years can have exposure to clinical EM activities.

The four SBL events were also created to fully engage learners in active learning as opposed to traditional didactic passive learning. Studies assessing the importance of active learning have shown that, after two weeks, students retain 90% of concepts they have “done” thru simulating an experience or participating in a real event, as opposed to only 20% of concepts they have heard and 10% of concepts they have read [17,18]. Retention is an important topic in medical education as several studies have shown that students have a propensity to quickly forget concepts [11]. Our data provide evidence that SBL may be an effective means to enhance medical student retention of important concepts.

In addition to providing exposure to clinical medicine, SBL events engage multiple learning styles. The four primary learning styles are aural, visual, reading/writing, and kinesthetic [19]. When these styles were assessed individually, the literature indicated the majority of medical students prefer kinesthetic styles [19]. Ironically, conventional lecture-based curricula commonly ignore kinesthetics, which makes the tactile approach of SBL both unique and of critical importance to student success. It is worth noting that when these learning styles were evaluated holistically, most students preferred bimodal learning, with kinesthetics commonly being one of these two preferred learning styles [19].

Our SBL events utilized a predominantly kinesthetic learning approach with aural and read/write learning styles included as well. While lectures may attempt to include multiple learning styles, they rely heavily on students' ability to learn from listening to a lecturer and reading bullet points of text on a screen. Kinesthetic learning involves students moving and physically manipulating the environment around them, which is the vast majority of what comprises SBL events [20]. The kinesthetic focus of SBL may be particularly beneficial, given that SBL can teach clinical knowledge that is not as effectively conveyed through traditional aural and visual didactic modalities.

Student enjoyment of this four-part EM-focused SBL curriculum is likely due to a variety of factors, including student interest in hands-on learning, especially in the context of the COVID-19 pandemic [21]. These SBL events provided students the opportunity to re-enter a clinical space (albeit a simulated one) and practice patient care, hands-on medical skills, and interprofessional communication via interactions with other students, standardized patients, and clinical instructors.

These educational events had several limitations, including a limited time frame and a small sample size. This study was not conducted past 90 days as the intention was to assess pre-clinical students within the same phase of training. This is a limitation as there was only one retention assessment for the mass casualty simulation and no retention assessments for the ultrasound simulation. The small sample size was due in part to pandemic-related gathering and social distancing requirements as well as limited resources (including simulation models, classrooms, and instructors). Additionally, the sample size was small because in order to accurately assess retention, students who did not attend previous events were not included in the calculations of retention for those events. Finally, students who attended these events had varying amounts of prior clinical experience, which could have potential effects on their pre-event knowledge, but this is a reality in all areas of medical education as students enter with varied backgrounds and previous healthcare experience.

## Conclusions

This study highlights an important way graduate education in healthcare can improve student exposure to clinical medicine outside of the classroom and encourage interdisciplinary collaboration between different healthcare roles. More importantly, this study provides evidence that SBL has the potential to address the issue of knowledge retention in pre-clinical students. Additionally, students enjoy SBL and remember clinical knowledge related to SBL events. Future studies should be conducted to further assess learners’ retention of clinical knowledge and technical skills acquired from SBL throughout their progression in medical training. The long-term impact of increased retention from SBL has the potential to shift the core methodology of preclinical and clinical medical education toward and increased reliance on SBL and other kinesthetic active learning modalities.

## Appendices

Suture lab instructions and evaluations

Instructions

Each student and instructor requires a suture kit containing a needle holder, toothed forceps, suture scissors, and two to three packs of Monocryl suture material. Each student and instructor will also need a simulated skin silicone pad or fruit including bananas and oranges.

Knowledge Evaluation Questions

(1) Which of the following is the thickest suture thread?

(2) What is the strongest suture type?

(3) Which of the following is the most appropriate suture to use for a wound on the face?

(4) Which suture type takes the longest to dissolve?

(5) What is the most common type of suture utilized in the ED?

(6) Which area is most amenable to repair with staples?

(7) Should a wound be washed out before it is closed?

(8) How long after you repair a facial laceration should you recommend the patient keep the sutures in before following up to have them removed?

MedEvac instructions and evaluation

Instructions

The following simulation equipment is required: airway task trainer with oropharyngeal airway, nasopharyngeal airway, laryngeal mask airway and bag valve masks, intraosseous task trainer, and bleed control task trainer with a tourniquet. After practicing with task trainers students should rotate thru the triage portion of the simulation with mannequins with moulage and injuries including compromised airway and partially amputated limbs during this part of the simulation students will hear the instructor read a scenario including vitals and pertinent details, students will then conduct a primary survey.

Triage scenario: [Both mannequins have amputated limbs and penetrating chest wounds to the back.] “You come across a helicopter crash with two individuals, the first patient has a blood pressure is 100/70, a heart rate of 110, and an RR of 0. Your second patient has a blood pressure of 95/70, a heart rate of 120, and an RR of 0." [Students should begin to use a bag valve mask and an airway adjunct on both patients, when they do this then say…]. “Your first patient remains apneic, your second patient now is breathing.” [Students should attempt to place a supraglottic device in patient one and tourniquets on both patients.]

Knowledge Evaluation Questions

(1) Which of the following is a common injection site for intraosseous needles?

(2) Intraosseous needles are used to inject fluids and/or medications into what part of the bone?

(3) When applying a tourniquet to an arm or leg, where should you place it?

(4) Utilizing mass casualty/disaster triage protocols, which of the following patients requires the most immediate medical attention?

(5) Which of the following criteria is NOT routinely utilized in disaster/mass casualty triage protocols?

(6) Which of the following is true regarding the medical response to civilian mass-casualty events?

ITEAM day instructions and evaluation

Instructions

These education events contain seven separate activities. Activities 1-6 should take 30 minutes each with Activity 7 taking about one hour.

Activity 1: Primary survey. Based on physician preference can be a verbal presentation or hands-on with high fidelity mannequin. Concepts covered will include assessment of the airway, C-spine, breathing and ventilation, circulation, traumas, burns, neurologic assessment, back wounds, and lacerations.

Activity 2: IO and IV placement. Requires intraosseous and intravenous task trainers.

Activity 3: Hemorrhage management. Requires bleed control task trainer.

Activity 4: Organophosphate decontamination scenario. Requires a high-fidelity mannequin with simulated urinary and bowel incontinence, diaphoresis, and miotic pupils as well as PPE for students to practice donning and doffing.

Activity 5: Airway. Requires airway task trainers include nasopharyngeal airway, oropharyngeal airway, bag valve masks, and laryngeal mask airways.

Activity 6: First five minutes of resuscitation. Requires a high-fidelity mannequin that has no pulse or cardiac activity when students enter the room.

Activity 7: Mass casualty simulation. Requires 10-20 standardized patients (and/or mannequins) with moulage. Each student will have a pack of green, yellow, red, or black tags to use. Students will be told before entering the room that there was a boiler explosion at a busy factory, and they need to triage with their fellow students because they are in a resource-limited location. Patient details including specific injuries will be provided if requested via email to , and mannequins should be utilized for pulseless/apneic patients as detailed. This event can be scaled down if required.

Knowledge Evaluation Questions

(1) Of the following airway devices, which is considered to be a “definitive” airway in an emergency setting, and will not require any further revision or manipulation?

(2) In approaching an adult patient with a suspected cervical spine injury who is also noted to have agonal respirations, what is the most appropriate method for opening the patient’s airway?

(3) Using START, what triage category would you assign the following patient in a mass-casualty scenario? A 46-year-old male who cannot move his legs, but who is awake, alert, and able to respond to commands. He is breathing 24 times per minute and has a radial pulse of 112.

(4) Using START, what triage category would you assign the following patient in a mass-casualty scenario? A 24-year-old female is found unconscious, with an obvious head wound. She is breathing spontaneously at a rate of 16 times per minute and has a radial pulse of 120.

(5) Once a patient has been assessed and triaged, their triage category (“tag color”) cannot change.

(6) A 32-year-old, otherwise healthy farm worker is brought into the emergency department via EMS after complaining of “feeling weird” while spraying insecticide. Which of the following symptoms would prompt you to immediately administer atropine and 2-PAM?

(7) Which of the following is incorrect with regard to blast injuries?

POCUS instructions and evaluations

Instructions

We advise having one standardized patient and one ultrasound device per two to three students present. Instructors will show students how to perform a trauma-focused exam in small two-to-three-person groups highlighting the pericardial view, perihepatic view, perisplenic view, suprapubic view, and thoracic view.

Knowledge Evaluation Questions

(1) Which probe is recommended when performing the FAST exam?

(2) Which probe is preferred to evaluate the lungs for pneumothorax and why?

(3) Which of the following are ultrasound diagnostic criteria for a AAA?

(4) The spine is _________ compared to the _________effusion.

(5) Which cardinal movement of the ultrasound probe would be the quickest way to check for free fluid above the right hemidiaphragm after assessing Morrison’s pouch?

(6) Where does fluid tend to accumulate first in a supine trauma patient with hemoperitoneum?
